# ‘I should have taken that further’ – missed opportunities during cardiovascular risk assessment in patients with psoriasis in UK primary care settings: a mixed‐methods study

**DOI:** 10.1111/hex.12404

**Published:** 2015-09-04

**Authors:** Pauline A. Nelson, Karen Kane, Anna Chisholm, Christina J. Pearce, Christopher Keyworth, Martin K. Rutter, Carolyn A. Chew‐Graham, Christopher E.M. Griffiths, Lis Cordingley, the IMPACT Team (Identification and Management of Psoriasis‐Associated Co‐morbidiTy)

**Affiliations:** ^1^ Manchester Centre for Dermatology ResearchInstitute of Inflammation & RepairUniversity of Manchester, and Manchester Academic Health Science CentreManchesterUK; ^2^ Manchester Centre for Dermatology and Manchester Centre for Health PsychologyUniversity of Manchester and Manchester Academic Health Science CentreManchesterUK; ^3^ The Endocrinology and Diabetes Research GroupInstitute of Human DevelopmentFaculty of Medical and Human SciencesUniversity of ManchesterManchesterUK; ^4^ Manchester Diabetes CentreCentral Manchester University Hospitals NHS Foundation Trust and Manchester Academic Health Science CentreManchesterUK; ^5^ Research Institute, Primary Care and Health SciencesKeele UniversityKeeleUK; ^6^ West Midlands CLAHRCKeeleUK; ^7^ Manchester Centre for Dermatology ResearchUniversity of Manchester and Manchester Academic Health Science CentreManchesterUK; ^8^ Salford Royal NHS Foundation TrustSalfordUK; ^9^ Manchester Centre for Health PsychologyUniversity of Manchester and Manchester Academic Health Science CentreManchesterUK

**Keywords:** cardiovascular disease, mixed methods, provider–patient communication, psoriasis, qualitative research

## Abstract

**Background:**

Unhealthy lifestyle is common in psoriasis, contributing to worsening disease and increased cardiovascular disease (CVD) risk. CVD risk communication should improve patients’ understanding of risk and risk‐reducing behaviours; however, the effectiveness of risk screening is debated and evaluation currently limited.

**Objective:**

To examine the process of assessing for and communicating about CVD risk in the context of psoriasis.

**Design:**

Mixed‐methods study in English general practices to (i) determine proportions of CVD risk factors among patients with psoriasis at risk assessment and (ii) examine patient and practitioner experiences of risk communication to identify salient ‘process’ issues. Audio recordings of consultations informed in‐depth interviews with patients and practitioners using tape‐assisted recall, analysed with framework analysis.

**Participants:**

Patients with psoriasis (*n* = 287) undergoing CVD risk assessment; 29 patients and 12 practitioners interviewed.

**Results:**

A high proportion of patients had risk factor levels apparent at risk assessment above NICE recommendations: very high waist circumference (52%), obesity (35%), raised blood pressure (29%), smoking (18%) and excess alcohol consumption (18%). There was little evidence of personalized discussion about CVD risk and behaviour change support in consultations. Professionals reported a lack of training in behaviour change, while patients wanted to discuss CVD risk/risk reduction and believed practitioners to be influential in supporting lifestyle management.

**Conclusions:**

Despite high levels of risk factors identified, opportunities may be missed in consultations to support patients with psoriasis to understand CVD risk/risk reduction. Practitioners need training in behaviour change techniques to capitalize on ‘teachable moments’ and increase the effectiveness of risk screening.

## Introduction

In the UK, the National Health Service (NHS) health check programme^1^ was established in 2009 to reduce the incidence of cardiovascular disease (CVD) and associated mortality, as well as inequalities in health.^2^ The programme offers CVD risk assessment to all individuals aged 40–74 years without existing CVD, diabetes or metabolic conditions, consisting of physiological measurements such as blood pressure (BP)/cholesterol as well as profiling of medical family history and lifestyle factors such as smoking. Guidelines state that individuals identified as having an elevated risk should be appropriately supported with lifestyle modification and/or pharmacological intervention to reduce such risk.^3^

### The health check programme debate

Health check attendance levels and treatment uptake have been lower than anticipated^4^ and evaluation in one large English health region found the checks failed to identify one‐third of people at high risk of developing diabetes.^2^ Krogsbøll *et al*.'s international Cochrane review^5^ suggests that health checks reduce neither morbidity nor mortality, but do increase the number of new diagnoses and pharmacological interventions, concluding that their benefits do not outweigh the associated harms. Others have also shown little change in reported prevalence of morbidity resulting from health checks.^6^ Subsequent debate among clinicians, researchers and policymakers about the effectiveness of health checks at the population level^7^ has led to calls to abandon them.^8^ Disadvantages of the programme include the following: low uptake,^9^ provision of inappropriate pharmacological solutions and false reassurance,^10^, ^11^ as well as discounting behavioural risk reduction strategies.^12^ However, the Cochrane review has been criticized for poor methodological quality,^13^ and other commentators stress the positive improvement in appropriate prescribing of statins following health checks.^14^, ^15^

Studies to evaluate the programme have so far been limited^9^, ^16^ and qualitative approaches to explore patient and practitioner experiences of health checks should form part of future evaluations.^17^

### Psoriasis and CVD risk

Psoriasis is a complex, long‐term inflammatory systemic condition, presenting with a skin rash, affecting at least 2% of the UK population.^18^ People with psoriasis often live long‐term^19^ with a range of challenging demands including high levels of chronic physical and psychosocial disability.^20^, ^21^ While severe psoriasis is associated with CVD,^22^, ^23^ the precise nature of this relationship is not yet fully understood.^24^

Unhealthy lifestyle behaviours such as excess alcohol consumption,^25^, ^26^ smoking,^27^, ^28^ inactivity and higher BMI^29^ are all known to be more common in people with psoriasis than the general population. These behaviours may have a role both in psoriasis onset and exacerbation or severity, as well as being risk factors for CVD.^28^, ^30^, ^31^, ^32^, ^33^, ^34^, ^35^ High levels of psychological distress associated with psoriasis^36^, ^37^ can increase risk behaviour and reduce motivation or capacity to engage in healthy behaviours.^38^ The National Institute for Health and Care Excellence (NICE) guideline for England and Wales on assessment and management of psoriasis^39^ recommends that practitioners identify and address comorbidities with patients, including discussion of lifestyle management.

### CVD risk communication and risk reduction

Perception of personal risk is highly complex and prone to systematic or error‐based biases.^40^, ^41^ The ways in which individuals perceive risk are influenced by their prior awareness and understanding of the risk as well as how it is presented to them.^42^ Communicating CVD risk is particularly complex because risk calculation/assessment involves amalgamating information about a range of physiological and lifestyle factors to predict patients’ future risk, often in the absence of current symptoms. Health professionals may use population risk values which lack personal relevance to patients,^43^ whereas personalized risk information may be required to increase risk understanding.^44^ Furthermore, the effect of communicating CVD risk upon the likelihood of engaging in risk reduction behaviours is unclear.

Qualitative research with healthcare practitioners shows that their personal attitudes towards/beliefs about health checks can be barriers to delivering lifestyle change support to patients^45^ and that practitioners’ skill sets could be enhanced by becoming confident and competent in the use of behaviour change techniques.^46^ Similarly, the literature suggests that patient barriers to engaging with and maintaining lifestyle modification include beliefs, emotions, information needs and social support.^47^, ^48^

This study aimed to explore patient and practitioner perspectives of the *process* of assessing for and communicating about CVD risk, using psoriasis as an exemplar of a condition where risk reduction in the form of lifestyle modification may be beneficial to disease management.

## Methods

The study involved two elements: (i) CVD risk factor assessment of adult patients with psoriasis above *and* below 40 years of age (to include younger patients not usually invited for a health check) in 13 general practices in North West England; and (ii) a ‘nested’ qualitative study of the process of risk communication in consultations for CVD risk assessment in people with psoriasis through in‐depth interviews with patients and practitioners, using excerpts from audio‐recorded risk communication consultations to assist questioning. People with psoriasis are not specifically targeted for CVD risk assessment; however, general practitioners (GPs) and practice nurses were asked to conduct risk assessment consultations in accordance with their usual health check procedures to capture routine practice.

### Sampling and recruitment

General practices varying in size and locality were identified and recruited from five primary care trusts across North West England. Practices were reimbursed for their participation. Using Read codes known to map to psoriasis and medications/topical preparations for psoriasis, practices identified patients with psoriasis over 18 years old on their lists. Those with severe mental health problems, without capacity to consent, the recently bereaved and the terminally ill were excluded by the GP. Identified patients were invited to attend a CVD risk assessment at their practice. Smaller practices mailed all eligible patients on their list; larger practices mailed an agreed number (depending on practice capacity), in which case a random number list was used to select potential participants.

Ethics approval was obtained from the North‐West Research Committee, Greater Manchester East (REC ref: 11/NW/0654). Patients expressing interest were telephoned by a researcher who explained the study and, with the patient's agreement, arranged a CVD risk assessment at their own practice. Consent to audio‐record the risk assessment and follow‐up consultations was sought from all participating practitioners and patients, with the aim of capturing as many recordings as possible across the sample. Participants whose consultations were recorded were sampled to undergo subsequent qualitative interviews on the basis of (i) consultations in which salient process issues were identified and (ii) diversity of personal characteristics. Practitioners were sampled to obtain a mix of GPs/practice nurses and patients for diversity on age and gender.

### Quantitative data (CVD risk assessment)

#### Data collection

On attendance at the practice, informed consent was acquired from patients and each completed a medical history questionnaire. The GP or practice nurse recorded a range of biomedical and behavioural measurements that are part of standard CVD risk screening procedures (see Table S1 for measurements recorded at risk assessment). Patients were recalled for a follow‐up discussion of their screening results if deemed necessary by the practice. The research team was informed if a follow‐up appointment was advised.

#### Data analysis

Risk factor variables were derived from measured or patient‐reported data based on current published UK guidelines.^49^, ^50^

### Qualitative data (CVD risk assessment processes)

#### Data collection: audio recordings and interviews

Consultations were audio‐recorded and used to inform subsequent in‐depth interviews with practitioners and patients about their experiences of risk communication during consultations using ‘tape‐assisted recall’ (T‐A‐R).^51^ The T‐A‐R approach enabled the interviewer to probe participants’ reflections through replaying excerpts from consultations to ground questioning in specific examples of risk communication or discussion of lifestyle management. Interviews with participants were conducted between August 2012 and November 2013 and guided by interview topic guides (see Table 1 for content of the interview schedules). Interviews were audio‐recorded, transcribed verbatim, anonymized and transferred to NVivo 10 for data management (QSR International Pty Ltd. Version 10, 2012).^52^

**Table 1:** Content of the interview schedules

Topics	
Patient interviews	Practitioner interviews
General questions	
Reasons for taking part in the study Understanding of the study/consultation purpose Understanding of CVD risk and link with psoriasis Perception of personal risk Any changes in views about health since talking part	Reasons for taking part in the study Understanding of the study Type and amount of information given to patients Strategies used to communicate risk to patients Techniques used to address patients’ lifestyle behaviour change Barriers/facilitators to doing lifestyle change work Training needs
Specific questions linked to recording excerpts	Specific questions linked to recording excerpts
Understanding of risk information conveyed by practitioner and ways to reduce risk Perceptions of sources of information, support for lifestyle behaviour change	Reflections on what was happening in the consultation including: aims, intentions, intended messages, techniques used, impressions of patients’ understanding, impressions of patients’ emotional reactions, use of personalized/general strategies

#### Data analysis: audio recordings and interviews

Two authors (AC and CP) familiarized themselves with the audio recordings to identify *process* issues (when and how CVD risk was discussed/addressed in the consultations) using a qualitative content analysis approach.^53^ An *a priori* analysis framework of topics relevant to CVD risk assessment guided critical listening/coding of audio recordings (see Table S2). Coding enabled identification of instances when biomedical and behavioural factors as well as additional factors pertinent to this specific patient group such as psoriasis severity, quality of life and mood were discussed. Additionally, by coding the audio recordings, practitioners’ communication techniques could be categorized in terms of (i) acknowledgement of patient cues for discussion, (ii) general communication style and (iii) approaches to addressing issues connected to risk/lifestyle. The two authors each analysed 50% of the audio recordings and discussed all coding to resolve and agree codes for ambiguous examples. The content analysis enabled identification of how CVD risk was addressed within consultations to guide selection of T‐A‐R excerpts to inform subsequent qualitative interviews.

Principles of framework analysis,^54^ developed for use in applied healthcare settings, were used to analyse the interview data. This approach facilitates investigation of pre‐set as well as emergent topics using constant comparison^55^ as data collection and analysis are conducted simultaneously until data saturation is achieved and no new insights are being generated. The research team met regularly to discuss key ideas emerging within and across interviews with particular attention to cases that differed from salient trends in the data and jointly agree a framework of main themes. Data were coded in NVivo enabling extraction of illustrative data.

## Results

### CVD risk assessment study

Thirteen general practices participated. Practice size (number of registered patients over age 18) varied between 1086 and 16 746 patients. Practices were located in a variety of rural and urban areas and varied in levels of deprivation. From 1446 invitations for CVD risk assessment sent, 447 people responded and 287 attended an appointment (220 aged 40 and over; 67 aged under 40), an overall attendance rate of 20%. Of those attending, 165 (57%) were female and the mean age was 53 years. Comparing those who did and did not attend, there was no difference in the proportion of women; however, a lower rate of attendance was recorded for the under 40s (15% compared with 25%, respectively).

Biomedical data were analysed for proportions of risk factors in the sample (see Table 2 for risk factor definitions) *evident at risk assessment* and thereby amenable to discussion during the consultation. Table 2 reports proportions of the sample with the following CVD risk factors at risk assessment: self‐reported smoking, self‐reported alcohol consumption, obesity, very high waist measurement and raised BP. The most common risk factors seen at the risk assessment were obesity (35%) and a very high waist measurement (52%) indicating central adiposity. At risk assessment, raised BP was found in 29% of those attending. This figure includes patients already prescribed antihypertensive medication (but not reaching treatment target) as well as those with previously unknown hypertension. Eighteen percentage of those assessed reported smoking and 18% reported drinking above the recommended units of alcohol per week.

**Table 2:** Risk factors identified at risk assessment

Risk factor	Definition of risk	%	Number with risk factor	Data reported (*N*)
Current smoker	Self‐reported smoker	18	52	283
Alcohol risk (units per week) > guidelines	Self‐reported units per week (males > 21; females > 14)	18	53	285
Raised blood pressure	Mean systolic (mm Hg) > 140 OR Mean diastolic (mm Hg) > 90 (final two of three readings)	29	84	287
Obesity	BMI (kg/m^2^) > 30	35	101	287
Very high waist circumference	Males (cm) > 102; females (cm) > 88	52	150	287

### Audio‐recorded consultations

Practitioners in 10 of the 13 participating practices agreed to consultation audio recording. A total of 130 CVD risk assessment and 15 follow‐up consultations were audio‐recorded (involving four GPs, nine practice nurses and 131 patients with psoriasis). Risk assessments were generally conducted in dedicated clinics making audio recording straightforward; however, follow‐up consultations were conducted on an *ad hoc* basis according to the practice's usual follow‐up procedure. As a consequence, the practices experienced major difficulties capturing follow‐up audio recordings.

Analysis of the audio‐recorded consultations revealed three core issues during patient–practitioner interactions. Firstly, there was *little evidence of detailed discussions* about CVD risk (core issue 1). When patients offered cues about concerns related to risk factors (e.g. consultation number 111: patient with high BP cues ‘…it's why I'm on this health kick – to get the weight down’), practitioners commonly responded by blocking or failing to pursue patients’ concerns, shutting down rather than opening up a dialogue. Secondly, practitioners demonstrated *a focus on recording information* rather than opportunistically addressing CVD risk reduction in consultations (core issue 2). This was evident in instances where a clear statement from the patient (e.g. consultation 101: ‘I'd love to lose a bit of weight to be honest’) elicited a disconnected practitioner response. Here the clinician appeared to be primarily fixed on documenting biomedical or behavioural data rather than incorporating the patient's agenda into the work of the consultation. Thirdly, *little evidence of skilled patient‐centred practice* was observed in consultations (core issue 3). Skilled practice was apparent in a minority (e.g. consultation 29: practice nurse, having determined the patient was not yet ready to stop smoking, closed the consultation with a prompt towards the possibility of future behaviour change ‘…so think about your smoking…you can either come to the GP or the smoking cessation clinic here, they'll accommodate you if you want some support with that’). This was a rare example of a practitioner recognizing and acting upon an opportunity to increase the salience of possible behaviour change in the patient's mind and support their self‐efficacy. This practice nurse was one of only two practitioners (both practice nurses) who consistently picked up on and responded to openings in consultations to address CVD risk with patients.

In summary, opportunities to discuss and address lifestyle behaviours such as smoking, diet and exercise which occur naturally in risk assessment consultations were often overlooked by practitioners in these recordings. Further extracts from the audio‐recorded consultations are presented in Table 3 as illustrative examples of instances where practitioners appeared to miss or use opportunities to address patient cues for discussion of CVD risk and/or lifestyle management.

**Table 3:** Consultation extracts – missed and used opportunities

Missed opportunities	
Extract	Interpretation (linked to core issues from consultation analysis)
*Extract 1 (Consultation 80)*	
***GP3:** So it works out at 42 units a week…*	
***Patient:** I'm just a normal bloke who goes to the pub on a Friday night that's all!*	Patient reports drinking above recommended weekly units of alcohol, uses humour to deflect discussion^a^
***GP3:** Yeah, yeah so it's all right, it's just that you know the rec… we just need to advise you what the current guidance is okay?*	Practitioner sidesteps discussion, falls back on standard recommendations rather than addressing individual need a ^,^ c
***Patient:** [Laughs]…[pause]*	No verbal response from patient^a^
***GP3:** Right now, let's have a look at your medications*.	GP changes topic to focus on recording medication use^a^^,^ b
*Extract 2 (Consultation 16)*	
***Patient:** I'm desperately trying to lose weight…*	While being weighed, patient gives clear cue about needing weight management support
***PN1:** That's 78.2 kilos ok [typing]…which comes to 12st 4Ibs*	Nurse focused on recording patient's weight b
***Patient:** [Gasps]*	Patient gives clear non‐verbal cue indicating emotional impact
***PN1:** See what you were before [typing]…more or less 5 kilos – a stone…*	Nurse does not respond to emotional cue and points out patient's weight gain^a^
***Patient:** Yeah…I put on a lot when I was first pregnant…*	Patient gives reason for weight gain
***PN1:** Yeah, 68 kilos there weren't you? Have you ever smoked?*	Nurse misses/blocks patient cue for discussion of weight and moves on to record smoking status, shuts down discussion a ^,^ b ^,^ c
*Extract 3 (Consultation 102)*	
***PN8:** We've got you down as 10 units per week [alcohol] last time*.	
***Patient:** Yeah I've cut it down last – er – I've been cutting down*.	Patient offers cue for possible discussion of own successful behaviour change
***PN8:** Right*	Nurse does not respond to patient cue, closes down discussion^a^^,^ c
***Patient:** I don't really drink…I'm trying to lose weight you see. Now if you'd have said psoriasis impacting weight, I'd have said that's what's caused all my weight problems since I was 7 [laughs] – I would honestly!*	Patient cues for discussion a second time, links own alcohol behaviour change with goal of weight loss and raises further link between psoriasis and weight
***PN8:** Right, um are you on any statins?*	Nurse fails to respond to specific concerns of patient, misses opportunity to discuss alcohol, weight and lifestyle–psoriasis links, closes down discussion, focuses on recording medication use^a^^,^ b ^,^ c

Used opportunities	
Extract	Interpretation (linked to core issues from consultation analysis – examples of *skilled practice)*
*Extract 1 (Consultation 30)*	
***PN2:** You are 13st 6lbs and it's saying at the most you should be 9st 11lbs you see on the computer, so…*	Nurse uses ‘guiding’ style and graphical representation of BMI categories to support patient's understanding of appropriate weight^e^
***Patient:** So I'm in the obese scale?*	Patient verbalizes concern about weight information provided
***PN2:** Yeah. So what we gonna do? What do you think you can do exercise wise? They say if you pick something that you like you will maintain it and keep doing it, you know what I mean? Rather than me saying to you ‘Right you need to go for a walk 5 times a week for half an hour’. You need to find something that you like doing really to maintain it and keep it going*	Nurse responds directly to patient's concern, signals that she will support patient, explores patient's confidence to make a change^d^^,^ e Gives rational for increasing/maintaining physical activity, encourages individualized choice^d^^,^ e Moves towards planning for change^e^
*Extract 2 (Consultation 61)*	
***PN9:** But first of all just as a first thought…is doing a food diary*	Nurse suggests a food diary as part of individual action planning with patient^d^^,^ e
***Patient:** Right yeah!*	Patient expresses interest in food diary activity
***PN9:** And actually writing down for a week what you have during the day and when you have it and absolutely writing everything down even if it's just a few crisps*	Nurse focuses on detail of food diary with patient^d^^,^ e
***Patient:** Even if it's just a few crisps or whatever, yeah, yeah course yeah*	Patient expresses interest in plan
***PN9:** Yeah, and then I can see you again with that and we have the option of referring you to the health trainer … we do have other referral procedures where we can actually send you to the Active Living Team… It's which fits into your lifestyle and which you would prefer to do?… If we could initially ask you to do a food diary then see me in a week to 10 days* with that?	Nurse offers review/follow‐up to discuss food diary, offers possibility of further options for more individualized support^d^^,^ e Encourages individualized choice^e^ Makes a plan with timeline for follow‐up^d^^,^ e
***Patient:** Food diary, of course, yep no problem*	Patient expresses interest in plan

a Core issue 1: lack of detailed CVD risk discussion.

b Core issue 2: practitioner focus on recording information.

c Core issue 3: lack of patient‐centred practice.

d Core issue 1: example of *skilled CVD risk discussion*.

e Core issue 3: example of *skilled patient‐centred practice*.

### Patient and practitioner interviews

In‐depth interviews were carried out with 29 patients (18 female; 12 < 40 years of age) and 12 practitioners (10 female; eight practice nurses and four GPs) who had consented to have their consultations audio‐recorded (see Table S3 for characteristics of interviewed patients and practitioners). Interviews focused on patient and practitioner perceptions of CVD risk assessment/follow‐up consultations. Excerpts from the audio‐recorded consultations, including examples of instances when opportunities for discussion of CVD risk and/or risk reduction appeared to have been missed, informed questioning.

Three key themes emerged from the analysis of the interviews: (i) limited shared discussion about CVD risk and lifestyle issues, (ii) limited provision of personalized risk reduction support to patients and (iii) the perceived influence of health practitioners in supporting risk reduction. These themes expand upon and illuminate the core issues (1, 2 and 3) revealed by the consultation data. Each is described below alongside relevant data extracts which highlight key contrasts in patients’ and practitioners’ views about the process of CVD risk assessment.

#### Theme 1: Limited shared discussion between patients and practitioners about CVD risk and lifestyle

Patients assumed that it was not their place to raise concerns in CVD risk assessment consultations because the process was essentially clinician‐ rather than patient‐driven. This led them to minimize the importance of risk and lifestyle‐related issues and contributed to the lack of discussion (core issue 1) apparent in consultations:

I don't want to go [to doctor] just because I'm worried about diabetes or I want advice about my weight…because they're so busy and a lot of things you can get on the internet so rather than waste an appointment you just browse. P12: Female, aged 45, obese, very high waist circumference

On hindsight I could've said [to the practitioner] ‘what would you recommend I do…to see if I can reduce any potential cardio risks?’ I wasn't sure how far I should go with that conversation – was it relevant? P9: Male, aged 38, obese, very high waist circumference

Furthermore, patients assumed that practitioners would view these issues as trivial and not a good use of their time. Some patients reported being too anxious to be able to raise health concerns with practitioners in the consultation and others seemed keen to preserve relationships with clinicians by not bothering them with ‘unimportant’ concerns:

They say at the end ‘have you got any questions?’ and then you've forgotten what's gone on previously…it's all hazy what's been said as well, you know? You're talking about your health so you are a bit apprehensive, forgetful. P29: Female, aged 33, no risk factors seen at assessment

Maybe [practitioners] can help you with weight loss and all that but I suspect there's a lot more people they need to help before they help me losing some weight! Ultimately, it's your own responsibility. P18: Male, aged 57; obese, very high waist circumference, smoker, drinks alcohol (declined to disclose quantity)

Practitioners reported that their main focus in these consultations was on gathering information from patients and informing/educating them about healthy lifestyle behaviours with reference to government recommendations or advice. This suggests that they were less focused on patient‐led discussion in which they could use cues in consultations to talk about patients’ own concerns and explains the focus on information recording and lack of detailed discussion apparent in the consultations (core issues 1 and 2):

From my point of view it was a data‐gathering exercise as opposed to a discussion about cardiovascular risk with the patient. Of course [patient] may have asked questions during that and if you had time you could try and answer. GP3

I ask them to eat oily fish three times a week – religiously. PN6

Practitioners described the consultations undertaken for the current study as similar or equivalent to the routine health checks they would normally conduct. The only differences identified were as follows: (i) The target group was atypical (adult patients with psoriasis of all ages); (ii) more time was allocated for consultations than in normal practice; and (iii) the consultation needed to be audio‐recorded:

It's not very different from doing a blood pressure clinic in terms of questions we need to ask, information we need to find out from patients, and then the advice that we give to them, so from that perspective it wasn't a big challenge. It's just the fact that things like the tape recorder, I think that puts a different slant on it. PN1

I saw it as more like a screening process really…like doing a well person's check, but the patient has psoriasis really. I do a lot of reviews anyway, like diabetic and heart reviews. So…to me it was a screening thing. PN2

Taken together, patients’ reluctance to instigate conversations with practitioners about risk‐related health concerns and practitioners’ focus on information gathering/advice giving meant that there was little shared discussion taking place during consultations that enabled patients to understand their risk status or ways to reduce risk. Practitioners reported that the conduct of the consultations differed little from that of customary health checks, suggesting that shared discussion may also lack prominence in routine practice.

#### Theme 2: Limited provision of personalized risk reduction support to patients

Practitioners reported that one of their roles is to provide standardized government recommendations and lifestyle advice to patients in CVD risk consultations:

I always treat it exactly the same. I don't change [information given]. It's just what I know is what I give out and that's it. That's as much as I do. PN3

I don't know whether [what was said] is conveying any message…but certainly [patient] knows what his waist size is. We haven't discussed anything further about that so it's difficult for me to guess [what patient understood]. GP2

There was limited evidence from practitioners’ accounts of tailored support being provided in response to specific information provided by individual patients, echoing the low level of skilled patient‐centred practice evident in consultations (core issue 3). It was notable that many practitioners expressed the view that their patients already possessed the knowledge required to understand and act appropriately to reduce CVD risk:

Most people know what they should and shouldn't be drinking, eating, exercising – shouldn't they? PN4

I think most people know where they're going wrong, they know that they shouldn't be eating three cakes every day…it's in the media all the time – get five a day in, take more exercise, eat less, move more – people know. PN7

Even when patients had several risk factors, they appeared not to prioritize discussion of them in the absence of current symptoms, with some seeing a need for action only once disease with perceptible symptoms had developed:

You tend to think that unless there's actually something wrong, they don't call you in. Well that's always been my belief. P8: Male, aged 54, obese, very high waist circumference, 24 units weekly alcohol

Until I develop cardiovascular disease, I'm not going to take much interest…if it happens I'll deal with it then. P3: Female, aged 64, obese, very high waist circumference

Some interpreted the lack of directive discussion by the practitioner as a signal *not* to change anything about their lifestyle, believing that the messages did not apply to them. This lack of individualized risk/lifestyle discussion in consultations (core issues 1 and 3) could lead patients to miss the personal relevance of health messages about CVD risk:

If [blood pressure] was high [PN] would probably say ‘well you need to look at eating this and cutting out that’. If there's nothing broken, then there's no need to fix it, is there? P23: Male, aged 32, overweight

Practitioners appeared to have a somewhat paradoxical standpoint in that they identified their role as providing standardized advice during CVD risk assessments, but at the same time, assumed patients already possessed the information required to make lifestyle changes. In contrast, patients often interpreted the absence of personalized engagement or advice from practitioners as a signal that the information was not relevant to or directed at them specifically, the implication being that they did not need to attend to risky health behaviours. This highlights the unintended consequences of the lack of detailed discussion and skilled patient‐centred practice found in the consultations (core issues 1 and 3).

#### Theme 3: The perceived influence of health practitioners in supporting risk reduction

Patients clearly perceived GPs and practice nurses as knowledgeable and competent both to help them understand CVD risk and make changes to lifestyle:

I thought – I'm taking [PN's] word for it that where I am is good for me. I'm going to believe it because she's in that position where she'd know. P11: Female, aged 52, obese, very high waist circumference

Some patients said they had been keen to discuss their risk and lifestyle with their practitioner but had not been given the opportunity within their consultation:

I thought [GP] might've asked me a bit more about what I was doing, how I was losing the weight, what exercise I was doing, but he didn't. P19: Female, aged 62, obese, very high waist circumference

Patients viewed primary care practitioners as approachable and trustworthy individuals with whom to appropriately discuss potentially sensitive concerns connected to body image or lifestyle behaviours, and perceived them to be in a position of influence in prompting their thinking on these issues:

I'd rather talk to a health practitioner than somebody at the gym who's stick thin, loads of muscles and a bit intimidating. So a nice normal average person who's got the information is a lot more comfortable. P12: Female, aged 45, obese, very high waist circumference

If a [health practitioner] says – ‘that's way too much mate, you shouldn't be having that much’ [alcohol], then you start thinking to yourself, hang on, he's a practitioner, I need to cut back a bit. P8: Male, aged 54, obese, very high waist circumference, 24 units weekly alcohol

In contrast to this patient perspective, practitioners (generally practice nurses, who, more than GPs, were tasked with risk assessment/reduction activity) reported views of themselves as lacking the power to effectively support patients with lifestyle changes. Nurses’ accounts highlighted a lack of confidence in broaching potentially sensitive behaviour change topics in case the patient–practitioner relationship was disrupted. In addition, they expressed pessimism about their degree of influence to motivate patients:

With hindsight I should have definitely taken that further [discussion with female patient drinking 30 units of alcohol per week]. I find that really interesting for reflective practice…I am too people pleasing. PN6

If they tell you they enjoy smoking I feel there's no point. They can't see the damage that's being done so they feel fine. You get ‘my granddad lived to be 102 and he smoked’. I can't say anything about that…you know that you're never going to get anywhere with them, so that's up to them. PN3

Furthermore, nurses suggested they were not well equipped to do such work, being unsure how to check patients’ understanding of risk/lifestyle issues. Only one nurse (PN9) had undergone specific, structured training in techniques to support lifestyle behaviour change. The remainder reported that a lack of training undermined their ability to undertake these tasks which they simultaneously identified as being key elements of their professional role. This offers insight into the lack of detailed discussion and skilled patient‐centred practice observed in the consultations (core issues 1 and 3):

I think we do need more training because it's difficult to get these things across. PN4

It's difficult to know what people understand because quite often they'll sit there and they'll agree with you and nod their head, but I don't know what they're thinking really. So I don't really know how else to deal with it. PN7

Patients viewed practitioners as being in a position to motivate them, to help them understand and address their CVD risk and to identify ways of reducing it. Moreover, they expressed the desire to address these issues with their GP or nurse. In contrast, GPs did not always perceive behaviour modification support as part of their role. Furthermore, practice nurses expressed very limited confidence to carry out these activities with patients. This means that patients are not benefitting from their attendance at CVD risk screening, a time at which the very data required to formulate a tailored risk reduction strategy is being collected and recorded by key healthcare professionals.

## Discussion

This mixed‐methods study of CVD risk factor assessment in people with psoriasis in UK primary care highlights important gaps in current practices around CVD risk screening. In particular, it demonstrates that CVD risk screening appears to be limited to a data collection activity rather than viewed as one component of a broader intervention strategy to reduce CVD risk. There was little evidence that opportunities for effective risk communication between patients and practitioners in consultations were recognized and acted upon by the practitioners conducting risk assessments. This study goes some way to offering a potential explanation for the debated ineffectiveness of national health check programmes and may explain Krogsbøll and colleagues’ findings^5^ that health checks can lead to an increased number of new diagnoses and pharmacological interventions while failing to reduce morbidity.

Our study shows that despite significant levels of risk factors identified in study participants at risk assessment (between one‐third and half classified as obese, with very high waist circumference and raised BP that would warrant further investigation and almost one‐fifth smoking and drinking over the recommended amounts), opportunities to support patients to understand CVD risk and/or identify risk reduction strategies may often be overlooked in consultations. A key explanatory factor was that practitioners’ confidence to deliver personalized lifestyle behaviour change support was low. While this study was undertaken in the context of trying to reduce psoriasis‐associated comorbidities, these findings have broader relevance to our understanding of the role of CVD screening and health checks.

A focus on the *processes* involved in CVD risk assessment at the level of the individual consultation provides crucial detail which aids our understanding of the apparently limited effectiveness of general health checks which have been identified at the population level^14^ as well as indicating direction for more effective preventive approaches.^56^ Missed opportunities in disease prevention have been previously identified in physicians’ practice,^57^, ^58^ but our study supports earlier literature which states that opportunities to address change are frequently presented in consultations.^59^, ^60^ Patients participating in the current study, as in other studies,^61^ wanted to discuss lifestyle issues with healthcare practitioners and furthermore expected it,^62^ and addressing unhealthy behaviours is recognized as a key element in the roles of health practitioners (e.g. healthy lives, healthy people;^63^ Making Every Contact Count initiative http://www.makingeverycontactcount.co.uk/; NICE public health behaviour change guidance).^64^ However, practitioners express low confidence in recognizing, acknowledging and intervening with patients to manage psoriasis and its comorbidities,^65^, ^66^, ^67^ and patients report inadequate understanding of psoriasis and perceptions of low control over the condition.^68^

Known factors which assist patient perception and understanding of risk could have been used by practitioners in this study to take advantage of opportunities presented in consultations such as helping people to understand *all* the different risk factors,^43^ capitalizing on the *trust* patients placed in them which influences responses to risk information^69^ and providing *personally relevant* information^43^, ^69^ as recommended in the key ‘personalized care’ health service policy.^70^ Additionally, CVD risk assessment consultations present particular opportunities for discussion of risk reduction strategies in the form of lifestyle behaviour change. Practitioners could incorporate lifestyle behaviour change techniques (which, when done well, are effective) into their practice.^71^, ^72^

### Strengths and limitations

A particular strength of this study was the use of T‐A‐R methods to go beyond self‐report and enable exploration of what practitioners do in practice. This addresses a limitation identified in previous studies of CVD risk communication in primary care.^73^ The study also has several limitations. Uptake of risk assessment was relatively low (possibly patients over 40 years of age had already been offered, and/or participated in a recent health check by their practice), and our sample may be unrepresentative of the wider population of people with psoriasis. The relatively small sample size means the risk factor proportions recorded may be imprecise in comparison with the general population with psoriasis. The audio‐recorded consultations may not reflect routine CVD risk assessment in practice; however, practitioners were asked to carry out the process of risk assessment according to their routine practice for CVD screening and they reported very little difference between the two. Additionally, it may be that only the most confident practitioners agreed to audio recording of their consultations, however, given the low level of skilled practice observed, this may be even lower in the ‘real world’. The small number of follow‐ups limits findings as practitioners may have taken opportunities to address issues of risk and lifestyle in consultations that were not captured by recordings. However, the audio recordings demonstrate that opportunities for intervention present themselves at the risk assessment consultation itself, and the approaches of two practitioners in particular show that such opportunities can be capitalized upon. Lastly, as practitioners and patients were in a therapeutic relationship, social desirability may have inhibited interview accounts.

### Practice implications

A shift in the focus of screening consultations is needed to encompass effective discussions and interventions to address CVD risk factors including behavioural ones. This means going beyond information/advice giving to change people's beliefs and increase motivation to make lifestyle changes and improve cardiovascular health.^43^, ^44^, ^74^ Practitioners could take advantage of the ‘teachable moment’, defined as opportunities presented for them to link people's health behaviours to current health status and estimated to occur naturally in 10% of doctor–patient consultations,^75^ capitalizing on these ‘cueing events’^76^ to prompt discussions about lifestyle change.^77^

## Conclusion

Screening for CVD risks is an activity which provides opportunities to engage patients in discussions about their current and future health status and offers practitioners the chance to provide timely brief interventions to improve future health. The benefits of health checks may not have materialized because of a focus upon data recording rather than intervention. Before abandoning health check programmes, it is important to attend to key *process* issues in risk assessment and encourage professionals to focus on helping patients understand the personal relevance of lifestyle behaviour choices, engage with the possibility of making changes and discuss individually appropriate strategies for change within the primary care consultation.

## Sources of funding

This article presents independent research funded by NIHR under its Programme Grants for Applied Research scheme (RP‐PG‐0608‐10163). The views expressed are those of the authors and not necessarily those of the National Health Service, the NIHR or the Department of Health. CEMG is an NIHR Senior Investigator. CCG is part‐funded by the National Institute for Health Research (NIHR) Collaborations for Leadership in Applied Health Research and Care West Midlands.

## Conflict of interests

No conflict of interests has been declared.

## Supporting information


**Table S1**. Measurements recorded at risk assessment.
**Table S2**. Consultation audio recordings: framework guiding critical listening/coding.
**Table S3**. Characteristics of patients and practitioners interviewed.

## References

[1] Department of Health . Putting Prevention First. NHS Health Check: Vascular Risk Assessment and Management Best Practice Guidance. London: Department of Health, 2009.

[2] Smith S , Waterall J , Burden ACF . An evaluation of the performance of the NHS Health Check programme in identifying people at high risk of developing type 2 diabetes. BMJ Open, 2013; 3: e002219. 10.1136/bmjopen-2012-002219 PMC3612750 23468469

[3] Department of Health . Putting Prevention First. Vascular Checks: Risk Assessment and Management. London: Department of Health, 2008.

[4] Cochrane T , Gidlow CJ , Kumar J , Mawby Y , Iqbal Z , Chambers RM . Cross‐sectional review of the response and treatment uptake from the NHS Health Checks programme in Stoke on Trent. Journal of Public Health, 2013; 35: 92–98. 23104892 10.1093/pubmed/fds088 PMC3580053

[5] Krogsbøll LT , Jørgensen KJ , Gøtzsche PC . General health checks in adults for reducing morbidity and mortality from disease. Journal of the American Medical Association, 2013; 309: 2489–2490.23780462

[6] Caley M , Chohan P , Hooper J , Wright N . The impact of NHS Health Checks on the prevalence of disease in general practices: a controlled study. British Journal of General Practice, 2014; 64: e516–e521. 25071065 10.3399/bjgp14X681013 PMC4111345

[7] Mant D . Health checks and screening: what works in general practice? British Journal of General Practice, 2014; 64: 493–494. 25267023 10.3399/bjgp14X681637 PMC4173700

[8] Krogsbøll LT , Jørgensen KJ , Gøtzsche PC . Universal health checks should be abandoned. British Medical Journal, 2013; 347: f5227. 24004991 10.1136/bmj.f5227

[9] Artac M , Dalton ARH , Babu H , Bates S , Millett C , Majeed A . Primary care and population factors associated with NHS Health Check coverage: a national cross‐sectional study. Journal of Public Health, 2013; 35: 431–439. 23881962 10.1093/pubmed/fdt069

[10] McCartney M . Too much medicine: where's the evidence for NHS health checks? British Medical Journal, 2013; 347: f5834. 24089427 10.1136/bmj.f5834

[11] Wookey SL . Universal health checks: making the dilemma of universal health checks clear to patients. British Medical Journal, 2013; 347: f5230. 24004992 10.1136/bmj.f5230

[12] MacAuley D . The value of conducting periodic health checks. British Medical Journal, 2012; 345: e7775. 23169871 10.1136/bmj.e7775

[13] Waterall J , Greaves F , Kearney M , Fenton KA . NHS Health Check: an innovative component of local adult health improvement and well‐being programmes in England. Journal of Public Health, 2015; 37: 177–184. 26022808 10.1093/pubmed/fdv062

[14] Artac M , Dalton ARH , Majeed A , Car J , Huckvale K , Millett C . Uptake of the NHS Health Check programme in an urban setting. Family Practice, 2013; 30: 426–435. 23377607 10.1093/fampra/cmt002 PMC3722503

[15] Soljak M , Majeed A , Millett C . Response to Krogsbøll and colleagues: NHS health checks or government by randomised controlled trial? BMJ, 2013; 347: f5984. 24108162 10.1136/bmj.f5984

[16] Jørgensen T , Kart Jacobsen R , Toft U , Aadahl M , Glümer C , Psinger C . Effect of screening and lifestyle counselling on incidence of ischaemic heart disease in general population: Inter99 randomised trial. British Medical Journal, 2014; 348: g3617. 24912589 10.1136/bmj.g3617 PMC4049194

[17] Chipchase L , Waterall J , Hill P . Understanding how the NHS Health Check works in practice. Practice Nursing, 2013; 13: 24–29.

[18] Parisi R , Symmons DPM , Griffiths CEM , Ashcroft DM , Identification M . Global epidemiology of psoriasis: a systematic review of incidence and prevalence. Journal of Investigative Dermatology, 2013; 133: 377–385. 23014338 10.1038/jid.2012.339

[19] Lebwohl M . Psoriasis. Lancet, 2003; 361: 1197–1204. 12686053 10.1016/S0140-6736(03)12954-6

[20] Anstey A , McAteer H , Kamath N , Percival F . Extending psychosocial assessment of patients with psoriasis in the UK, using a self‐rated, web‐based survey. Clinical and Experimental Dermatology, 2012; 37: 735–740. 22998542 10.1111/j.1365-2230.2012.04457.x

[21] de Korte J , Sprangers MAG , Mombers FMC , Bos JD . Quality of life in patients with psoriasis: a systematic literature review. Journal of Investigative Dermatology Symposium Proceedings, 2004; 9: 140–147. 10.1046/j.1087-0024.2003.09110.x 15083781

[22] Gelfand JM , Neimann AL , Shin DB , Wang X , Margolis DJ , Troxel AB . Risk of myocardial infarction in patients with psoriasis. Journal of the American Medical Association, 2006; 296: 1735–1741. 17032986 10.1001/jama.296.14.1735

[23] Mehta NN , Azfar RS , Shin DB , Neimanns AL , Troxel AB , Gelfand JM . Patients with severe psoriasis are at increased risk of cardiovascular mortality: cohort study using the General Practice Research Database. European Heart Journal, 2010; 31: 1000–1006. 20037179 10.1093/eurheartj/ehp567 PMC2894736

[24] Samarasekera EJ , Neilson JM , Warren RB , Parnham J , Smith CH . Incidence of cardiovascular disease in individuals with psoriasis: a systematic review and meta‐analysis. Journal of Investigative Dermatology, 2013; 133: 2340–2346. 23528816 10.1038/jid.2013.149

[25] Kirby B , Richards HL , Mason DL , Fortune DG , Main CJ , Griffiths CEM . Alcohol consumption and psychological distress in patients with psoriasis. British Journal of Dermatology, 2008; 158: 138–140. 17999698 10.1111/j.1365-2133.2007.08299.x

[26] Sommer DM , Jenisch S , Suchan M , Christophers E , Weichenthal M . Increased prevalence of the metabolic syndrome in patients with moderate to severe psoriasis. Archives of Dermatological Research, 2006; 298: 321–328. 17021763 10.1007/s00403-006-0703-z

[27] Favato G . High incidence of smoking habit in psoriatic patients. American Journal of Medicine, 2008; 121: e11. 10.1016/j.amjmed.2007.12.007 18374672

[28] Fortes C , Mastroeni S , Leffondre K *et al* Relationship between smoking and the clinical severity of psoriasis. Archives of Dermatology, 2005; 141: 1580–1584. 16365261 10.1001/archderm.141.12.1580

[29] Naldi L , Chatenoud L , Linder D *et al* Cigarette smoking, body mass index, and stressful life events as risk factors for psoriasis: results from an Italian case‐control study. Journal of Investigative Dermatology, 2005; 125: 61–67. 15982303 10.1111/j.0022-202X.2005.23681.x

[30] Finucane MM , Stevens GA , Cowan MJ *et al* National, regional, and global trends in body‐mass index since 1980: systematic analysis of health examination surveys and epidemiological studies with 960 country‐years and 9.1 million participants. Lancet, 2011; 377: 557–567. 21295846 10.1016/S0140-6736(10)62037-5 PMC4472365

[31] Gerdes S , Zahl VA , Weichenthal M , Mrowietz U . Smoking and alcohol intake in severely affected patients with psoriasis in Germany. Dermatology, 2010; 220: 38–43. 19996578 10.1159/000265557

[32] Poikolainen K , Karvonen J , Pukkala E . Excess mortality related to alcohol and smoking among hospital‐treated patients with psoriasis. Archives of Dermatology, 1999; 135: 1490–1493. 10606054 10.1001/archderm.135.12.1490

[33] Setty AR , Curhan G , Choi HK . Smoking and the risk of psoriasis in women: Nurses’ Health Study II. American Journal of Medicine, 2007; 120: 953–959. 17976422 10.1016/j.amjmed.2007.06.020 PMC2696351

[34] Smith KE , Fenske NA . Cutaneous manifestations of alcohol abuse. Journal of the American Academy of Dermatology, 2000; 43: 1–16. 10863217 10.1067/mjd.2000.104512

[35] Wolk K , Mallbris L , Larsson P , Rosenblad A , Vingard E , Stahle M . Excessive body weight and smoking associates with a high risk of onset of plaque psoriasis. Acta Dermato‐Venereologica, 2009; 89: 492–497. 19734975 10.2340/00015555-0711

[36] Baker CS , Foley PA , Braue A . Psoriasis uncovered – measuring burden of disease impact in a survey of Australians with psoriasis. Australasian Journal of Dermatology, 2013; 54: 1–6. 10.1111/ajd.12010 23379483

[37] Kimball AB , Gieler U , Linder D , Sampogna F , Warren RB , Augustin M . Psoriasis: is the impairment to a patient's life cumulative? Journal of the European Academy of Dermatology and Venereology, 2010; 24: 989–1004. 20477920 10.1111/j.1468-3083.2010.03705.x

[38] Poikolainen K , Reunala T , Karvonen J , Lauharanta J , Karkkainen P . Alcohol intake – a risk factor for psoriasis in young and middle‐aged men. British Medical Journal, 1990; 300: 780–783. 1969757 10.1136/bmj.300.6727.780 PMC1662565

[39] National Institute for Health and Care Excellence . Psoriasis: The Assessment and Management of Psoriasis (CG153). London: NICE, 2012.

[40] Kahneman D , Tversky A . Prospect theory: an analysis of decision under risk. Econometrica, 1979; 47: 263–291.

[41] Weinstein ND . Why it won't happen to me: perceptions of risk factors and susceptibility. Health Psychology, 1984; 3: 431. 6536498 10.1037//0278-6133.3.5.431

[42] Edwards A , Elwyn G . Understanding risk and lessons for clinical risk communication about treatment preferences. Quality in Health Care, 2001; 10: I9–I13. 11533431 10.1136/qhc.0100009.. PMC1765742

[43] Waldron C‐A , van der Weijden T , Ludt S , Gallacher J , Elwyn G . What are effective strategies to communicate cardiovascular risk information to patients? A systematic review. Patient Education and Counseling, 2011; 82: 169–181. 20471766 10.1016/j.pec.2010.04.014

[44] Ahmed H , Naik G , Willoughby H , Edwards AGK . Communicating risk. British Medical Journal, 2012; 344: e3996. 22709962 10.1136/bmj.e3996

[45] Honey S , Bryant LD , Murray J , Hill K , House A . Differences in the perceived role of the healthcare provider in delivering vascular health checks: a Q methodology study. BMC Family Practice, 2013; 14: 172. 24229342 10.1186/1471-2296-14-172 PMC3870972

[46] Gudzune KA , Clark JM , Appel LJ , Bennett WL . Primary care providers’ communication with patients during weight counseling: a focus group study. Patient Education and Counseling, 2012; 89: 152–157. 22819710 10.1016/j.pec.2012.06.033 PMC3462265

[47] Murray J , Craigs CL , Hill KM , Honey S , House A . A systematic review of patient reported factors associated with uptake and completion of cardiovascular lifestyle behaviour change. BMC Cardiovascular Disorders, 2012; 12: 120. 23216627 10.1186/1471-2261-12-120 PMC3522009

[48] Murray J , Fenton G , Honey S , Bara AC , Hill KM , House A . A qualitative synthesis of factors influencing maintenance of lifestyle behaviour change in individuals with high cardiovascular risk. BMC Cardiovascular Disorders, 2013; 13, doi:10.1186/1471‐2261‐13‐48. 10.1186/1471-2261-13-48 PMC3716917 23829636

[49] National Institute for Clinical Excellence . Obesity: Guidance on the Prevention, Identification, Assessment and Management of Overweight and Obesity in Adults and Children (CG 43). London, 2006: https://www.nice.org.uk/guidance/cg43. 22497033

[50] National Institute for Clinical Excellence . Hypertension: The Clinical Management of Primary Hypertension in Adults (CG 127). London, 2011: http://www.nice.org.uk/guidance/cg127.

[51] Cape J , Geyer C , Barker C *et al* Facilitating understanding of mental health problems in GP consultations: a qualitative study using tape‐assisted‐recall. British Journal of General Practice., 2010; 60: 837–845. 20939945 10.3399/bjgp10X532567 PMC2965968

[52] Richards L . Using NVivo in Qualitative Research. Thousand Oaks, CA: Sage, 1999.

[53] Schreier M . Qualitative Content Analysis in Practice. London, Thousand Oaks, New Delhi, Singapore: Sage Publications Ltd, 2012.

[54] Ritchie J , Lewis J . Qualitative Research Practice: A Guide for Social Science Students and Researchers. London, Thousand Oaks, New Delhi: Sage, 2003.

[55] Corbin J , Strauss A . Basics of Qualitative Research: Techniques and Procedures for Developing Grounded Theory. Thousand Oaks, New Delhi, London, Singapore: Sage Publications, 2008.

[56] Karwalajtys T , Kaczorowski J . An integrated approach to preventing cardiovascular disease: community‐based approaches, health system initiatives, and public health policy. Risk Management and Healthcare Policy, 2010; 3: 39–48. 22312217 10.2147/RMHP.S7528 PMC3270919

[57] Ma J , Urizar GG , Alehegn T , Stafford RS . Diet and physical activity counseling during ambulatory care visits in the United States. Preventive Medicine, 2004; 39: 815–822. 15351551 10.1016/j.ypmed.2004.03.006

[58] Stange KC , Flocke SA , Goodwin MA , Kelly RB , Zyzanski SJ . Direct observation of rates of preventive service delivery in community family practice. Preventive Medicine, 2000; 31: 167–176. 10938218 10.1006/pmed.2000.0700

[59] Flocke SA , Stange KC , Goodwin MA . Patient and visit characteristics associated with opportunistic preventive services delivery. Journal of Family Practice, 1998; 47: 202–208. 9752372

[60] Stange KC , Flocke SA , Goodwin MA . Opportunistic preventive services delivery – are time limitations and patient satisfaction barriers? Journal of Family Practice, 1998; 46: 419–424. 9598000

[61] McPhail S , Schippers M . An evolving perspective on physical activity counselling by medical professionals. BMC Family Practice, 2012; 13: 31. 22524484 10.1186/1471-2296-13-31 PMC3438055

[62] Duaso MJ , Cheung P . Health promotion and lifestyle advice in a general practice: what do patients think? Journal of Advanced Nursing, 2002; 39: 472–479. 12175356 10.1046/j.1365-2648.2002.02312.x

[63] HM Government . Healthy Lives, Healthy People: Our Strategy for Public Health in England. London: The Stationery Office, 2010.

[64] National Institute for Health and Care Excellence . Behaviour Change: Individual Approaches (PH 49). London: NICE, 2014.

[65] Nelson PA , Barker Z , Griffiths CEM , Cordingley L , Chew‐Graham CA , Team I . ‘On the surface’: a qualitative study of GPs’ and patients’ perspectives on psoriasis. BMC Family Practice, 2013; 14: 158. 24138455 10.1186/1471-2296-14-158 PMC3828399

[66] Nelson PA , Keyworth C , Chisholm A *et al* ‘In someone's clinic but not in *mine*’ – practitioners’ views of supporting lifestyle behaviour change with patients with psoriasis: a qualitative study. British Journal of Dermatology, 2014; 171: 1116–1122. 24981809 10.1111/bjd.13231

[67] Richards HL , Fortune DG , Weidmann A , Sweeney SKT , Griffiths CEM . Detection of psychological distress in patients with psoriasis: low consensus between dermatologist and patient. British Journal of Dermatology, 2004; 151: 1227–1233. 15606519 10.1111/j.1365-2133.2004.06221.x

[68] Nelson PA , Chew‐Graham CA , Griffiths CEM , Cordingley L , Team I . Recognition of need in health care consultations: a qualitative study of people with psoriasis. British Journal of Dermatology, 2013; 168: 354–361. 22880951 10.1111/j.1365-2133.2012.11217.x

[69] Alaszewski A , Horlick‐Jones T . How can doctors communicate information about risk more effectively? British Medical Journal, 2003; 327: 728–731. 14512483 10.1136/bmj.327.7417.728 PMC200810

[70] Department of Health . Skills for Care, Skills for Health: Common Core Principles to Support Self Care: A Guide to Implementation. London: Department of Health, 2008.

[71] Estabrooks PA , Nelson CC , Xu S *et al* The frequency and behavioral outcomes of goal choices in the self‐management of diabetes. The Diabetes Educator, 2005; 31: 391–400. 15919639 10.1177/0145721705276578

[72] Greaves CJ , Sheppard KE , Abraham C *et al* Systematic review of reviews of intervention components associated with increased effectiveness in dietary and physical activity interventions. BMC Public Health, 2011; 11: 119. 21333011 10.1186/1471-2458-11-119 PMC3048531

[73] Bonner C , Jansen J , McKinn S *et al* Communicating cardiovascular disease risk: an interview study of General Practitioners’ use of absolute risk within tailored communication strategies. BMC Family Practice, 2014; 15: 106. 24885409 10.1186/1471-2296-15-106 PMC4042137

[74] Rollnick S , Kinnersley P , Stott N . Methods of helping patients with behaviour change. British Medical Journal, 1993; 307: 188–190. 8343750 10.1136/bmj.307.6897.188 PMC1678361

[75] Cohen DJ , Clark EC , Lawson PJ , Casucci BA , Flocke SA . Identifying teachable moments for health behavior counseling in primary care. Patient Education and Counseling, 2011; 85: e8–e15. 21183305 10.1016/j.pec.2010.11.009 PMC4389220

[76] McBride CM , Emmons KM , Lipkus IM . Understanding the potential of teachable moments: the case of smoking cessation. Health Education Research, 2003; 18: 156–170. 12729175 10.1093/her/18.2.156

[77] Flocke SA , Stange KC . Direct observation and patient recall of health behavior advice. Preventive Medicine, 2004; 38: 343–349. 14766118 10.1016/j.ypmed.2003.11.004

