# Supplementary material for: ‘I should have taken that further’ – missed opportunities during cardiovascular risk assessment in patients with psoriasis in UK primary care settings: a mixed‐methods study
Source: Health Expect. 2015 Sep 4;19(5):1121–37. doi: 10.1111/hex.12404 (PMC5053232; doi:10.1111/hex.12404)
Supplement: Supplementary file 1 — Table S1. Measurements recorded at risk assessment. Table S2. Consultation audio recordings: framework guiding critical listening/coding. Table S3. Characteristics of patients and practitioners interviewed. [file HEX-19-1121-s001.docx]

**SUPPORTING INFORMATION**

**Table 4. Measurements recorded at risk assessment**

| **ITEMS RECORDED** |
| --- |
| 1. Smoking status and alcohol units consumed per week (patient-reported) 2. Height and weight 3. Hip and waist circumferences 4. Sitting blood pressure three times 5. Blood samples* to be analysed at the local hospital biochemistry department for:    - glycosylated haemoglobin (HbA_1c_)    - fasting lipids (total cholesterol, LDL-cholesterol, HDL-cholesterol and triglycerides)    - fasting glucose    - liver and renal function   *In some cases the blood sample had been taken in advance of the assessment appointment |

**Table 5. Consultation audio-recordings: framework guiding critical listening/coding**

| **TOPICS** |
| --- |
| Biomedical factors   1. Blood pressure 2. Cholesterol 3. Diabetes risk 4. Family history of CVD 5. Waist and hip measurements 6. Weight and BMI 7. Mood (including stress, depression, anxiety) 8. Psoriasis (severity, co-morbidities, associations with lifestyle behaviours/mood)   Lifestyle behavioural factors:   1. Smoking 2. Alcohol consumption 3. Physical activity 4. Diet   Practitioner communication factors   1. Acknowledgment of patient cues for discussion (e.g. practitioner acknowledges, explores, ignores, shuts down, changes the topic) 2. General communication style (e.g. fast-versus slow-paced, serious versus light-hearted) 3. Approaches to addressing CVD risk (e.g. amount and type of risk information feedback, offer of management plan or review of CVD risk (for lifestyle behaviours or other CVD risk factors) |

**Table 6. Characteristics of patients and practitioners interviewed**

| **PATIENTS** | **Female** | **Male** | **Totals** |
| --- | --- | --- | --- |
|  | 18 | 11 | **29** |
| **Age**  <40  ≥40 | 7  11 | 5  6 | **12**  **17** |
| **PRACTITIONERS** | **Female** | **Male** | **Totals** |
|  | 10 | 2 | **12** |
| **Professional discipline**  Practice Nurse (PN)  General Practitioner (GP) | 8  2 | 0  2 | **8**  **4** |
